# The contribution of Ghanaian patients to the reporting of adverse drug reactions: a quantitative and qualitative study

**DOI:** 10.1186/s12889-018-6285-9

**Published:** 2018-12-18

**Authors:** Tom G. Jacobs, H. Hilda Ampadu, Jarno Hoekman, Alexander N. O. Dodoo, Aukje K. Mantel-Teeuwisse

**Affiliations:** 1 WHO Collaborating Centre for Pharmaceutical Policy and Regulation, Division of Pharmacoepidemiology and Clinical Pharmacology, Utrecht Institute for Pharmaceutical Sciences UIPS), Utrecht University, Utrecht, the Netherlands; 2 The African Collaborating Centre for Pharmacovigilance, Accra, Ghana; 3 Innovation Studies Group, Copernicus Institute for Sustainable Development, Utrecht University, Utrecht, the Netherlands

**Keywords:** Pharmacovigilance, Patient reporting, Adverse drug reactions, Communication, Questionnaire, Patient interviews

## Abstract

**Background:**

Under-reporting of adverse drug reactions (ADRs) is a major challenge for pharmacovigilance in Africa. This study sets out to assess the level of awareness of Ghanaian patients about ADRs and ADR-reporting and explores how different patients in Ghana recognize an ADR and the steps they take when they experience an ADR.

**Methods:**

This was a two-part study consisting of a survey to quantify the awareness of Ghanaian patients on ADRs and ADR-reporting, and in-depth interviews to explore how patients recognize an ADR and the steps they take thereafter. Participants were selected from 28 health care facilities (HCF) in rural and urban areas in 4 out of the 10 administrative regions of Ghana. Chi-square tests were used to examine associations between demographic variables and i) awareness of ADRs and ADR-reporting, ii) ADR experience and iii) awareness of the Ghana Food and Drug Authority (Ghana-FDA) and its patient reporting system (PRS). Only participants that indicated they experienced an ADR were included for the in-depth interviews. Data was investigated for participants’ awareness of ADRs, ADR reporting and steps taken when they experience ADRs.

**Results:**

Of the total 572 participants enrolled in the study, 14% indicated they were unaware of ADRs and were excluded. Of the remaining 491 participants, 38% had experienced an ADR, of which 67% reported the ADR, 68% of them reported it to a doctor. Only 3% of the 491 participants were aware of the Ghana-FDA’s PRS. The interview phase consisted of 33 patients who had experienced an ADR. Three key findings from the interview phase were; most participants recognized an ADR themselves, the symptoms of the ADR were the most mentioned reason for reporting and participants experienced a wide variety of obstacles in ADR-reporting.

**Conclusions:**

Most Ghanaian patients appear unaware of or unable/unwilling to use formal national channels for ADR reporting like the Ghana-FDA PRS. Motivation for ADR reporting appeared mainly personal and not communal. These findings warrant further attention in order to increase patient reporting of ADRs.

## Background

Spontaneous reporting of Adverse Drug Reactions (ADRs) is the cornerstone of pharmacovigilance. ADRs continue to be a major public health issue as they are a major cause of patient morbidity and mortality [1]. The costs associated with treatment of ADRs are an economic burden on resource-limited health care systems such as those in most African countries [2].

An important aim of pharmacovigilance is the detection of signals by timely sharing of data on ADRs to identify previously unknown medicines-related safety issues. Per the World Health Organization’s (WHO) definition, an ADR is “a response to a drug which is noxious and unintended, and which occurs at doses normally used in man for the prophylaxis, diagnosis, or therapy of disease, or for the modification of physiological function” [3]. Worldwide, under-reporting of ADRs is a major challenge for successful pharmacovigilance [4]. Under-reporting is particularly problematic in Africa and is well documented l [5–8]. Individual case safety reports (ICSRs) from Africa to the WHO International Database - VigiBase™ is less than1% of the global total even though Africa has 15% of the World’s population [5]. Several studies have been carried out to explore the high under-reporting in Africa compared to other regions. Most of these studies focus on under-reporting by health care workers (HCWs) [9–11].

To address the issue of under-reporting, some countries in Africa, e.g. Ghana and Kenya, have embarked on patient reporting initiatives [12, 13]. Patient reporting is generally seen as a positive development for pharmacovigilance [14]. In the Netherlands, for example, patient reporting has been shown to increase the number of reported ADRs and also provides a new perspective on the experiences of ADRs [15]. Whilst data from the Netherlands and other high-income countries cannot necessarily be translated to Africa, it is encouraging to notice the efforts made by national pharmacovigilance centres in Africa to promote direct patient reporting as a means of overcoming chronic under-reporting.

For patient reporting to work however, it is important for patients to be aware of ADRs and the formal national channels for reporting ADRs and to be able to recognize an ADR. They must be able to easily use these channels and should find value in using them. There is paucity of data on patients’ awareness of ADRs in Africa and even more limited data on direct patient reporting of ADRs in Africa. There is also little understanding of how patients identify ADRs and what they do when they experience an ADR.

ADR reporting awareness campaigns in sub-Saharan Africa (SSA) countries typically focus on HCWs and rarely on patients. However, it is patients who experience ADRs and are able to give a first-hand account of what they have experienced making them an integral part of any ADR reporting process [16]. A study in the Netherlands concluded that the severity of the ADR and the need to share experiences were the main reasons why patients reported ADRs [17]. Research in Portugal showed that patients were more likely to spontaneously report ADRs which are severe or when they were worried about the symptoms of the ADR [18]. These findings, however, cannot be wholly extrapolated to SSA because of major differences in health care delivery systems, accessibility of HCWs, awareness of ADRs and health care regulations [7]. Moreover, there are differences in levels of education, culture and living conditions amongst people in Ghana and other SSA countries compared to those living in Europe and other high-income countries. Such differences may lead to variations in knowledge and perception on medications, ADRs and ADR reporting [19, 20]. It is also of importance to know what motivates patients in Ghana to report an ADR and whether they know the formal channels for ADR reporting including direct patient reporting. A recent study by Sabblah et al. on patients’ perspectives on ADR reporting in Ghana concluded that there is high patient awareness (82%) of the national pharmacovigilance centre and relatively high ability to report (50%) [26]. The work by Sabblah et al., however, took place in only 2 pharmacies (out of the national total > 15,000 pharmacies and other licensed dispensers of medicines) and consisted of investigator-administered structured questionnaires. This limits the generalisability of the findings towards the whole country, but it shows the importance that researchers are attaching to patient reporting of ADRs. We therefore set out to find the potential contribution of Ghanaian patients to the ADR reporting process by identifying the quantum of reporting by patients and their awareness of the various channels for direct patient reporting of ADRs. To build upon the work of Sabblah et al., our study involved 28 facilities in 4 administrative regions - 40% of the administrative areas of Ghana including rural and urban areas - to ensure stronger external validity. Our study aimed to quantify the awareness of Ghanaian patients on ADRs and ADR reporting and explore how patients in Ghana recognize an ADR and the steps they take after experiencing an ADR by using mixed methods.

## Methods

This is a two-part study involving both quantitative and qualitative approaches. The first part consisted of a survey to quantify the awareness of Ghanaian patients on ADRs and ADR-reporting. The second, qualitative part consisted of one-on-one in-depth interviews to explore how Ghanaian patients recognize an ADR and the steps they take when they experience an ADR.

### Selection of participants

Participants were selected from 28 health care facilities (HCF) in rural and urban areas in 4 out of 10 administrative regions of Ghana (Ashanti, Greater Accra, Eastern and Central regions). The HCF included government hospital pharmacies, private hospital pharmacies, community pharmacies and licensed Over The Counter (OTC) medicine sellers also known as “chemical sellers” to cover the full Ghanaian drug delivery system. Participants reflected multiple local language groups and were randomly selected after being supplied medication at a pharmacy or dispensary. They had to be at least 18 years and speak English, Twi, Ga or Fante. The researchers aimed to include an average of 20 participants per HCF to have an indicative sample of the population from the different facilities. So, the total targeted sample size was 560 participants.

Participants who indicated they had experienced an ADR in the survey phase were eligible for enrolment into the interview phase. Participants were selected by means of the maximum variation sampling strategy in order to obtain data from a wide range of patients [22]. The factors considered in the sampling strategy included gender, age, educational attainment, severity of experienced ADRs, whether or not the ADR was reported and rural/urban area of living. Data analysis started after conducting 20 interviews and the selection process continued until no new themes or categories emerged from the final four interviews (data saturation).

### Data collection and analysis

All surveys and in-depth interviews were conducted between November 2016 and December 2016.

#### Survey

In addition to the collection of demographic information, our survey included 7 questions and 5 sub-questions about the participants’ awareness of ADRs, their reporting behaviours and the information provided by the pharmacy or dispensary on possible ADRs to the dispensed medicines [Additional file 1 available upon request]. Two trained research assistants and the lead investigator (TJ) conducted all surveys. Upon being dispensed a medication at the pharmacy or dispensary, the researchers approached the potential participant. The rational of the study was explained, verbal informed consent sought and if participant agreed, they were enrolled into the study. The survey was piloted twice, respectively on 3 and 10 participants with different demographics. The pilots led to some changes in the formulation of the questions. The data from the pilots were not included in the analysis.

Chi-square tests were used to compare the demographic variables and i) awareness of ADRs and ADR-reporting, ii) whether participants had experienced ADRs and iii) awareness of participants on the Ghana Food and Drug Authority (Ghana-FDA) and its patient reporting system (PRS). Additionally, the Mantel-Haenszel test for trend was performed to check for differences in awareness of ADRs and ADR reporting in groups of patients with different age ranges and educational levels. Statistical Package for the Social Sciences (SPSS) software version 24 was used for all statistical analyses.

#### In-depth interviews

**Fig. 1:**
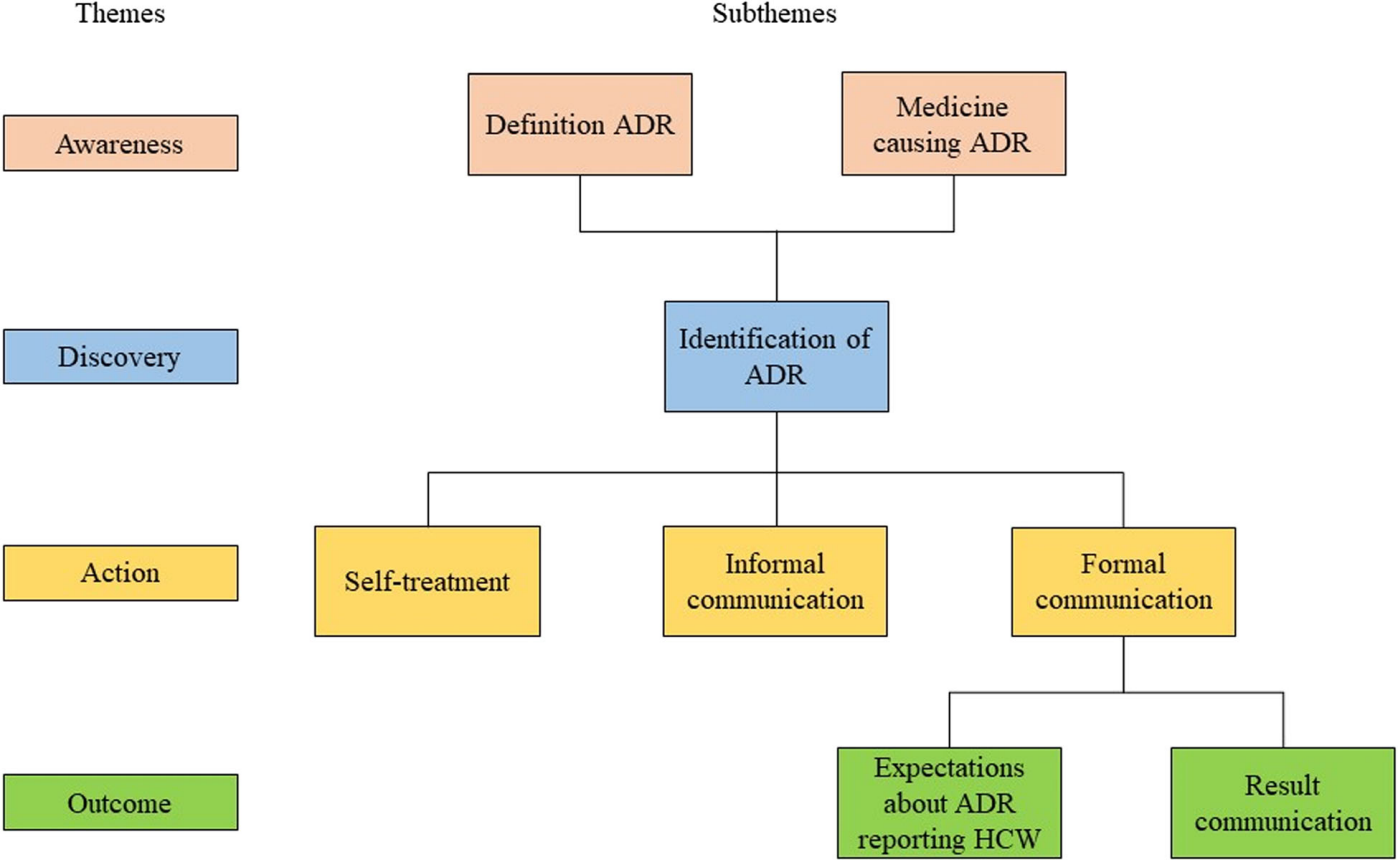
Conceptual framework used in the qualitative data analysis. ADR = adverse drug reaction, HCW = health care worker

A concise guideline was developed for the in-depth interviews. The guideline consisted of an introduction and 7 main questions on the four themes in the Conceptual Framework below (Fig. 1).

The way patients discover ADRs (Discovery) and reasons why they act when experiencing an ADR (Action) were two themes within the conceptual framework based on earlier studies [17, 18, 21, 23]. These earlier studies focussed on the themes separately, mostly with a quantitative methodology and in high-income countries. Our study used a qualitative methodology to provide a better understanding of all the steps patients take when they experience ADRs. Awareness of ADRs (Awareness) was a theme deduced from the surveys. Most sub-themes as well as the fourth theme about the outcome of reporting an ADR (Outcome) emerged inductively from the interview data. This theme included expectations of the patient about further actions taken by the HCW with the report of the ADR and the result of the consult with the HCW. The interview guideline underwent some minor changes after the first 4 interviews and reflects these four themes and their subthemes [Additional file 2 available upon request].

The interviews took place at the participant’s home or place of work to make them feel more comfortable and free to speak. Prior to the interview, written consent was sought. The consent form was read to participants with low literacy and they signed with a thumbprint. The lead investigator conducted all in-depth interviews in the presence of a translator. The interviewer asked follow-up questions if necessary for clarification. Each interview ended with the interviewer providing the participant the opportunity to speak freely about the issues discussed. Each participant was interviewed once with the interviews ranging between 10 and 20 min. All interviews were electronically recorded and transcribed verbatim by the lead investigator. Two research assistants assisted in transcribing the interviews conducted in local languages.

Data analysis of the interviews was conducted by the qualitative content analysis process using the themes awareness, discovery, action and outcome as outlined by Bengtsson [24] and Elo and Kyngas [25]. The analysis was both a deductive and inductive process in that the data was investigated for a priori issues relating to the objective but also captured unanticipated explanations and patterns. The inductive approach was particularly important to identify new actions and reasons to act after experiencing an ADR. Awareness, action and discovery were used as preformulated leading themes. Initial reading of 20 randomly selected transcripts was done by the lead investigator, units of meaning of the themes in sentences or paragraphs were highlighted and explanations of why it was important were noted. The highlighted units of meaning were then abbreviated in codes. These codes were grouped into categories within subthemes of the conceptual framework (Fig. 1) or new subthemes that emerged. A more experienced research team member (HHA) also coded the 20 randomly selected transcripts and both sets of codes were compared. The process of rereading transcripts and updating the framework continued until no further modifications were needed. This updated framework was used for the second coding process. In this process, 10 interviews were read and coded by four research team members together at the same time. The remaining 23 transcripts were read and coded by the researchers individually and discussed afterwards. All codes were substantiated with quotes and explanations, which were used to identify and interpret patterns in the data. The NVivo software program (version 11) was used to assist the analysis.

#### Ethical approval

The research protocol was reviewed and approved by the Committee on Human Research, Publications and Ethics of the Kwame Nkrumah University of Science and Technology (KNUST), reference number: CHRPE/AP/481/16.

### Survey results

A total of 572 participants were enrolled in the study. However only 571 surveys were analysed because one survey was completed incorrectly. The demographic characteristics of the participants are shown in Table 1.

**Table 1:** Demographics of all participants from the survey and the in-depth interviews compared to the numbers population of Ghana from 2010 [38]. JSS = Junior Secondary School, JHS = Junior High School, SSS = Senior Secondary School, SHS = Senior High School

		Survey		In-depth interviews		Figures Ghana
Variable		N	%	N	%	%
Gender						
	Female	293	51,3%	13	42%	52,8%
	Male	278	48,7%	18	58%	47,2%
Age						
	18–24	70	12,3%	1	3%	24,7%*
	25-34	127	22,2%	8	26%	27,3%
	35–44	117	20,5%	6	19%	19,1%
	45–54	108	18,9%	7	23%	13,0%
	55–64	76	13,3%	6	19%	7,3%
	65–74	54	9,5%	3	10%	4,7%
	75 ≤	19	3,3%	0	0%	3,8%
Education**						
	None	56	9,8%	3	10%	28,5%
	Primary/JSS/JHS	191	33,5%	8	26%	34,1%
	Secondary/SHS/SSS	173	30,3%	10	32%	27,3%
	Tertiary	149	26,1%	10	32%	10,2%
	Unknown	2	0,3%	–	–	–
Area of living						
	Rural area	140	24,5%	8	26%	49,1%
	Urban area	431	75,5%	23	74%	50,9%

*age group 15–19 was used, corrected to age range 18–19 and added to age group 20–24

**The figures from the educational attainment in Ghana and the four regions are from people 15 ≤ years old

In this study, 14% of participants (*n* = 80) indicated they had never heard of the words “side effect”, “adverse drug reaction” or their local equivalents. These participants were excluded from answering any further questions in the survey. Of the remaining 491 participants who completed the survey, majority were aware of ADRs and that it was possible to report these ADRs. However, examination of the data indicated that there was limited awareness of the formal ADR reporting system in Ghana as shown in Table 2. Only 45 of the 491 participants had heard of the PRS and of these only 16 (36%) indicated they knew how to report an ADR via the PRS, meaning only 3% of the total population that finished the survey knew how to report an ADR using the PRS. Moreover, only 0.5% of all participants who knew where to report, indicated they would report directly to the Ghana FDA, while 68% of them would report to a doctor. Of 439 participants that received medicines from the dispensary, 6% received information about possible side effects of medicines from their HCW.

**Table 2:** Persons or institutions to whom the participants reported or would report an adverse drug reaction (ADR). FDA = Ghana Food and Drug Authority, MoH = Ministry of Health

	Responses		Gender	Age	Education	Area of living
Are you familiar with the word side effect or ADR? *N = 571*	Yes	491 *(86,0%)*	*p* = 0.329	*p* = 0.004* T = 0.014*	*p* = < 0.001* T = < 0.001*	*p* = 0.038*
	No	80 *(14,0%)*				
Did you ever experience an ADR? *N = 491*	Yes	186 *(37,9%)*	*p* = 0.849	*p* = 0.018* T = 0.624	p = < 0.001* T = < 0.001*	*p* = 0.084
	No	305 *(62,1%)*				
Did you report the ADR? *N = 186*	Yes	123 *(67,2%)*	*p* = 0.963	*p* = 0.451 *T* = 0.066	*p* = 0.424 *T* = 0.475	*p* = 0.322
	No	60 *(32,8%)*				
Do you know where to report? *N = 491*	Yes	425 *(86,6%)*	*p* = 0.913	*p* = 0.817 *T* = 0.891	*p* = 0.097 *T* = 0.033	*p* = 0.231
	No	66 *(13,4%)*				
Do you know the organisation responsible for collecting reports of ADRs? *N = 491*	Yes	33 *(6,7%)*	*p* = 0.664	*p* = 0.704 *T* = 0.527	p = < 0.001* T = < 0.001*	*p* = 0.017*
	No	458 *(93,7%)*				
Are you familiar with the PRS? *N = 491*	Yes	45 *(9,2%)*	*p* = 0.162	*p* = 0.238 *T* = 0.159	*p* = < 0.001* T = < 0.001*	*p* = 0.047*
	No	446 *(90,8%)*				
Information about ADRs provided by pharmacists *N = 439*	Yes	25 *(5,7%)*				
	No	414 *(94,3%)*				

*indicates statistical significance

Participants with higher education were significantly more likely to be familiar with the words “side effect”, “ADR” or their local translation (*p* < 0.001). Additionally, they were more likely to be familiar with the organisation that is responsible for ADR reporting (Ghana FDA) and the PRS (*p* < 0.001). Participants with a higher education level also experienced an ADR more frequently (*p* < 0.001). Older participants were significantly more likely to be familiar with the words “side effect”, “ADR” or their local translation (*p* = 0.004) compared to younger participants. Similarly, participants living in urban areas were more likely to be familiar with these words (*p* = 0.038) than participants living in rural areas. They were also more likely to be familiar with the organisation that is responsible for ADR reporting (*p* = 0.017) and the PRS (*p* = 0.047).

### In-depth interview results

In total, 33 participants were enrolled in the interviews (Table 1). Two interviews were excluded from the analysis leaving only 31; one participant did not understand the questions and could not answer them, and a second participant had a very different explanation of an ADR.

#### Awareness

Participants were asked to define an ADR in their own words. Most participants (*n* = 21) described an ADR as a negative reaction after taking a medicine. Other participants mentioned every effect after taking a medicine (*n* = 4), an unexpected effect after taking a medicine (*n* = 3), the effect after taking an overdose of a medicine (n = 2) or the medicine is not working (*n* = 1) as definitions of an ADR. For example, one participant described an ADR as: *“If you should take the drug and should become plenty, it gives you a side effect.”* [Participant 7: male, finished primary school]. Secondly, patients were asked if they knew the name of the medicine that caused the ADR. Half of the participants did not know the name of the medicine; most of them mentioned that they forgot (*n* = 4) or just didn’t know the name of the medicine (*n* = 9). Two participants indicated they did not know the medicine that caused the ADR, because they took multiple drugs: *“If I was taking one particular medicine, I would say ‘okay when I take this medicine, this is the side effect’. But when you are taking combined drugs, taking about 4-5 different types of drugs, you cannot tell. There is no way you can tell.”* [Participant 26: male, finished tertiary education].

#### Discovery

Patients were asked how they knew they were experiencing an ADR. Most participants (*n* = 26) assessed the ADR themselves. They could relate the medicine to the symptoms they were experiencing without the help of a second person. Most of them related the ADR in time fashion (or temporally) with a certain medicine (*n* = 23). Also, some participants read the patient information leaflet or did research on the internet. The temporal assessment of an ADR is illustrated by a quote: *“The way I was before I visited the hospital, it has become over (after taking the medicine).”* [Participant 11: female, no education].

Only one participant assessed an ADR with the help of a family member. The remaining 4 participants had their ADRs assessed by a HCW; a doctor because they thought the ADRs were a disease: *“The person (general practitioner) checked my folder (hospital dossier) and realized that the drug I was given, gave me the reaction.”* [Participant 4: female, finished secondary education].

#### Action

In-line with this theme, the researchers first asked what the patient did when they experienced an ADR and what motivated them to do something about it. Most of them took multiple actions after experiencing the ADR. Based on the data, three main possible actions were deduced: self-treatment, informal communication and formal communication.

For self-treatment, most participants (*n* = 14) stopped taking the medicine without or before consulting someone. One participant mentioned he reduced the dose of the medicine, one started taking other medicines for the ADR and three tried to minimize the symptoms of the ADR. An example: *“Water is good, so I take water, always taking more water so that the thing (the ADR) can come out. So that is what I did.”* [Participant 25: female, finished secondary education]. The other participants (*n* = 11) did not self-treat.

We defined “informal communication” as communication with other patients, family members or friends. Most participants mentioned they discussed the ADR with family members (*n* = 21) and friends (*n* = 9). Some participants were advised by a relative or friend to visit a HCW or to be careful with the medication. Others instructed and educated family members or friends about the ADR. They often advised them not to take that same medicine (*n* = 8). For example, one participant said *“when I see somebody I say: be careful when you take that drug. That, I think, is the best I could do.”* [Participant 17, male, finished tertiary education]. Another action that could be distinguished within informal communication was the communication with fellow sufferers who experienced the same ADR (*n* = 5). They mainly advised each other not to buy or take the medicine in question again. Finally, some participants had no informal communication (*n* = 3) and in two of the interviews, the participants did not mention having informal communication.

The third action, formal communication, included participants that reported their ADR through formal channels. None of the participants had reported an ADR to the PRS. They reported their ADRs to a doctor (*n* = 19), a pharmacist (*n* = 3), a medical inspector (*n* = 1) or a nurse (n = 1). They reported to a specific HCW because they prescribed the medicine that caused the ADR. Participants also mentioned they visited a doctor rather than a pharmacy, because they have more faith in the knowledge of the doctor. For example, a participant who visited the doctor mentioned: *“Because they (doctors) have a lot of information.”* [Participant 10: female, finished tertiary education]. One participant mentioned that she went to a specific doctor, because her health insurance only covered for that doctor. One participant went to a doctor because her mother worked at that HCF as a nurse, and she did not want to report the ADR out of shame.

When an ADR was reported, the patients were asked why they reported it. Participants mentioned multiple motives to report an ADR. However, none of them described communal motivations such as contributing data to the reporting system or the knowledge based on ADRs. Most participants (*n* = 16) reported because of the symptoms they were experiencing. Those participants either wanted to treat the symptoms of the ADR, thought their symptoms were caused by another disease or the initial disease was not cured yet. Other participants were seeking for more information about the ADR (*n* = 4). These motives are driven by the wellbeing of the participant itself. Other participants were advised by other people to visit a HCW (n = 4), wanted to complain about the medicine (*n* = 4) or wanted to complain about the HCW who prescribed the medicine (*n* = 2). An example: *“I went there (the pharmacy) with an expectation, because I want her (the head pharmacist) to know that some of the people she is working with are not competent or don’t know their work. For that matter, that is going to bring a lot of effects on us.”* [Participant 9: male, finished primary education]. One participant mentioned that she was taught that she had to see a HCW every time she experienced an ADR. She said: *“So if we take a medicine and it is not good, we must come back here (the hospital).”* [Participant 11: female, no education]. These motives were focussed mainly on personal benefit.

According to the interviews 7 participants did not have any formal communication, some of them were not aware they could visit a HCW with an ADR, others doubted the capability of the HCW, did not want to bother the HCW or thought the distance to the health care facility was too far. Some participants also did not want to visit the HCW because the self-treatment was successful already or the medicine that caused the ADR was not prescribed or bought from a pharmacy in a health care facility. An example: *“Oh, because I didn’t buy the medicine from them (pharmacy/hospital) so I can’t go and report there.”* [Participant 13: male, finished tertiary education].

#### Outcome

If a patient reported an ADR through a formal channel, they were asked about the outcome of their reporting. Some of the outcomes from formal communication were; the HCW changed the medicine that caused the ADR (*n* = 17), gave an additional prescription (*n* = 4) or did nothing (*n* = 4). In the 19 interviews where participants’ expectations on ADR-reporting or follow-up were discussed when talking about outcomes, only one participant thought the HCW wrote down the ADR in his medical folder. The other participants were not certain what the HCW did with their report (*n* = 9), mostly because the HCW did not communicate with them. For example, one participant said: *“Because at times if they (doctors) give you some drugs, you take it and then you feel something (ADR). Next time when you go there and tell them, they just hear you, but they will not say anything.”* [Participant 32: female, finished primary school]. The remaining participants thought the doctor did not do anything (n = 9) with their report. One participant said: *“You go to a doctor (to report an ADR), he takes his money and end of story.”* [Participant 20: male, finished secondary school].

## Discussion

This study aimed to quantify the awareness of Ghanaian patients on ADRs and ADR-reporting and to explore how patients recognize an ADR and the steps they take thereafter. A key finding from the survey was that of the 491 participants, 38% had experienced an ADR of which 67% reported the ADR. Of these 68% reported it to a doctor. Overall, only 3% were aware of the Ghana-FDA’s PRS. Participants with higher education were more likely to have experienced an ADR whereas participants with higher education or living in urban areas or both were significantly more likely to be aware of ADRs and the PRS. Three key findings from the interview phase were that most participants recognized an ADR themselves, the symptoms of the ADR were the most mentioned reason for reporting and participants experienced a wide variety of obstacles in ADR-reporting.

The results from our survey differ considerably from that published by Sabblah et al. who found high patient awareness (82%) of the National Pharmacovigilance Centre and relatively high awareness of the possibility to report directly to the centre (50%) [26]. The different outcomes could be attributed to study design as well as the immediate effect of an FDA-Ghana radio and TV campaign to promote ADR-reporting in June 2016 [26]. Our data showed that patients with better awareness of ADRs and those who reported ADRs more often had higher education and more frequently lived in an urban area, which is in line with other findings in literature [18]. In low and middle-income countries, especially those with growing economies, disparity throughout the country may be higher than in high-income countries [27] and particularly health literacy may differ substantially between regions [28]. Spatial patterns of ADR-reporting may reflect this inequality. The observed differences between the two studies therefore highlight the importance of ensuring wide and diverse coverage of facilities when undertaking such studies, although this comes with associated high cost.

The conclusions from the survey were further explained with interviews. The survey revealed that the majority of participants were aware of ADRs and could report these ADRs. However, the interviews revealed some participants who were aware of the ADRs but did not know the name of the medicines that caused the ADR. The name of the medicine is one of the four mandatory fields that must be completed on an ADR-reporting form according to the Council for International Organizations of Medical Sciences (CIOMS) [29]. Therefore, not knowing the name of the suspected medicine(s) makes it impossible to report an ADR. It appears that the inability to recall the names of the medicines is linked to the dispensing practices in Ghana, as in other resource-limited countries. In Ghana it is a common practice to dispense from bulk, patients are typically given medications in a small white envelope most times not labelled as was also observed by our research team. This provides further evidence supporting previous studies which concluded that issues in dispensing medicines in SSA included poor labelling of dispensed medicines from bulk, poor patient counselling, dispensing by non-pharmacists, less qualified personnel and illiteracy as well as presence of products with labels in other languages apart from the official national languages [30]. This is supported by the finding that almost no patient indicated to have received any information about ADRs in this study. Follow-up studies could further investigate the dispensing practices with regard to information provision about ADRs.

In discussing motivations for reporting, a large percentage of participants (67%) indicated they reported the ADR. However, the interviews unearthed that the reasons to report an ADR were mostly driven by personal benefits. These reasons differ considerably from patients in high-income countries whose reasons for reporting were mainly driven by communal motives [31]. This can be explained by the substandard information provision by HCWs and the fact that most participants were unaware of the PRS. The communal motives of reporting can only be realised when patients get feedback on the effects of patient reporting and are aware of the PRS and its functioning, e.g. through accumulation of ADRs leading to evidence generation on causality. This will help patients appreciate the fact that reporting is not only for their own benefit but also for the benefits of others. Also, the survey data indicated that only few pharmacists in Ghana provided information about ADRs of the medicines administered to patients compared to other countries [32, 33]. The authorities and HCWs concerned need to let patients appreciate the reasons why they must report ADRs and the contribution to public health.

The survey and interviews revealed several obstacles to ADR-reporting. In Table 3 all identified obstacles are summarized and potential solutions are suggested. Lax regulatory enforcements appear to play a key role in low ADR reporting. It is a well-known fact in Ghana that medicines can be purchased from anywhere such as in buses, open market and from individuals in addition to the regulated licenced premises. It is estimated that 10–20% of all medicines is obtained illegally, but there are no confirmatory data [34]. Unlicensed sellers are mainly driven by financial incentives and are typically not properly educated about ADRs [32], thus are unlikely to provide any information on ADRs or how to report them. Purchasing medicines from unlicensed and itinerant sellers makes it difficult for patients to report ADRs because sometimes they are reluctant to mention or cannot trace where or whom they bought their medicines from as the interviews revealed.

**Table 3:** Identified obstacles experienced in ADR-reporting by Ghanaian patients and possible solutions to it

Obstacle	Potential solutions
Poor dispensing practices of medicines	Improve the regulation of medicine dispensing practices. Urging pharmaceutical companies to produce smaller medicine boxes Educate HCWs on good dispensary practices of medicines in their education program and by in-service training.
Substandard recognition of ADRs by patients	HCWs and primary schools should focus on educating (lower educated) patients on ADRs and how to recognize and assess ADRs. An easy tool can be developed to assist patients in the recognition and assessment of ADRs.
Skipping the first line of healthcare in reporting ADRs	The authorities concerned need to make patients more aware of avenues to report and particularly urge patients to report ADRs to their first line of care which is the pharmacy attendants and then other HCWs. Pharmacists or attendants in turn need to improve their participation in ADR-reporting by improving their patient engagement with the hope of establishing a lasting trust-based relationship.
Lacking awareness to report ADRs to HCWs	Better information provision practices from HCWs by including ADR reporting/patient education in the curriculum of healthcare disciplines Targeted campaigns by the Ghana-FDA.
Socio-economic differences between patients and HCWs	Point out alternative options for patient reporting of ADRs such as their first line of care (pharmacist) and/or the PRS.
Lacking awareness of the PRS and willingness to use it	Campaigns to make patients aware of the PRS Creating a patient-friendly version of the PRS

The second identified obstacle is substandard recognition of ADRs. It appears that participants in the in-depth interviews mostly assessed ADRs themselves which corresponds with other research [21]. The process of recognizing and assessing an ADR adequately is difficult and requires a lot of knowledge and can lead to substandard recognition of ADRs [23]. The knowledge gap as revealed by this study can contribute to the low rate of lower educated people that experienced (or recognized) an ADR compared to higher educated people in this study and compared patients in high-income countries [33, 35].

The first line of care for Ghanaian patients is the pharmacist and pharmacists are more likely to report an ADR when they see one compared to doctors [36]. Hence it is of major concern to see from both the survey and in-depth interviews in this study that patients will rather report their ADRs to doctors and not pharmacist/pharmacy attendants.

The survey revealed that Ghanaian patients lack awareness of the PRS and moreover lack the willingness to use it which is a major obstacle in ADR reporting. Also, not being aware of the possibility of reporting an ADR to a HCW emerged in the in-depth interviews as a reason not to report. Finally, being afraid to bother the doctor with an ADR was mentioned in one in-depth interview. Others also indicated that the high socio-economic status of the doctor is a challenging factor in the patient-doctor relation.

Based on these findings, we recommend the Ghana Food and Drug Authority to continue their education and awareness creation about ADRs but also target awareness creation to areas outside the capital cities and use medium of communication that citizen’s living in these areas are familiar with. Further, it is important not to settle on one dominant route for ADR reporting but keep the system flexible and allow for different ways of reporting depending on patient needs and geographical contexts. We suggest that an emphasis on the benefits of patient reporting and on different routes to facilitate such reporting should form part of all awareness campaigns.

This study is the first of its kind to obtain data on the behaviour of Ghanaian patients when they experience ADR from the patient’s perspective using mixed methods. Moreover, the population in both parts of the study was heterogeneous and representing 4 administrative regions of Ghana including the 2 most populous regions – Greater Accra and Ashanti regions. Also, participants in rural areas were included. More highly educated participants, elderly participants and participants living in urban areas were included in this study compared to the overall Ghanaian demographics. An explanation for this is that patients who use medicines that are distributed by official HCFs is not a proper reflection of the general population of the country. Apart from that, most HCFs in Ghana are located in urban areas [27] and the rural-urban migration makes it difficult to determine if someone lived in an urban or rural area [37]. The population sample of the survey covers the full formal Ghanaian health care delivery system but excludes data from patients who buy medicines from unlicensed medicine sellers, since these were not included in the study. A limitation of the study is that only patients that had experienced an ADR were included in the qualitative part of the study. It can be assumed that these participants had more knowledge about ADRs compared to participants that did not experience an ADR before. Also, participants who were not aware of the existence of ADRs were not asked any further questions in the survey. This could have led to an overestimation of the number of patients that experienced an ADR and patients that are aware of the PRS. Another limitation is the possibility of receiving socially desirable answers from the participants. However, the researchers tried to prevent this by asking open and neutral questions and not telling the participants too much about the aim of the study.

## Conclusions

Most Ghanaian patients are aware of ADRs, but especially participants that are older, low educated and live in rural areas seem less likely to be aware of ADR. Moreover, lower educated patients seem to fall short on recognizing ADRs. Incidence of ADR-reporting to HCWs is high among Ghanaian patients. However, most of them appear unaware of or unable/unwilling to use formal national channels for ADR reporting like the Ghana-FDA PRS. Patients appear driven by personal benefit in reporting ADRs instead of communal benefit which may be due to low awareness of the PRS. There are multiple obstacles that hamper patient reporting of ADRs in Ghana which warrant further attention to increase patient reporting of ADRs. Further studies on information provision about ADRs and ADR reporting by medicine dispensers and the impact of different regulatory measures on the patients’ knowledge of ADR reporting and the PRS could help overcome some of these obstacles.

## Abbreviations

ADR: Adverse Drug Reaction
FDA: Food and Drug Authority
HCF: Health Care Facility
HCW: Health Care Worker
KNUST: Kwame Nkrumah University of Science and Technology
MAH: Market Authorization Holder
MoH: Ministry of Health
PRS: Patient Reporting System
SPSS: Statistical Package for the Social Sciences
SSA: Sub-Saharan Africa
WHO: World Health Organization
